# Genetic distance and ancestry proportion modify the association between maternal genetic risk score of type 2 diabetes and fetal growth

**DOI:** 10.1186/s40246-024-00645-1

**Published:** 2024-07-19

**Authors:** Tesfa Dejenie Habtewold, Prabhavi Wijesiriwardhana, Richard J. Biedrzycki, Fasil Tekola-Ayele

**Affiliations:** 1 Epidemiology Branch, Division of Population Health Research, Division of Intramural Research, Eunice Kennedy Shriver National Institute of Child Health and Human Development, National Institutes of Health, 6710B Rockledge Drive, Bethesda, MD 20892-7004 USA; 2 Glotech, Inc., contractor for Division of Population Health Research, Division of Intramural Research, Eunice Kennedy Shriver National Institute of Child Health and Human Development, National Institutes of Health, 6710B Rockledge Drive, Bethesda, MD 20892-7004 USA

**Keywords:** Birthweight, Fetal weight, Fetal growth, Type 2 diabetes mellitus, Genetic ancestry proportion, Genetic distance, Genetic similarity, Genetic risk score

## Abstract

**Background:**

Maternal genetic risk of type 2 diabetes (T2D) has been associated with fetal growth, but the influence of genetic ancestry is not yet fully understood. We aimed to investigate the influence of genetic distance (GD) and genetic ancestry proportion (GAP) on the association of maternal genetic risk score of T2D (GRS_T2D_) with fetal weight and birthweight.

**Methods:**

Multi-ancestral pregnant women (*n* = 1,837) from the NICHD Fetal Growth Studies – Singletons cohort were included in the current analyses. Fetal weight (in grams, g) was estimated from ultrasound measurements of fetal biometry, and birthweight (g) was measured at delivery. GRS_*T2D*_ was calculated using T2D-associated variants identified in the latest trans-ancestral genome-wide association study and was categorized into quartiles. GD and GAP were estimated using genotype data of four reference populations. GD was categorized into closest, middle, and farthest tertiles, and GAP was categorized as highest, medium, and lowest. Linear regression analyses were performed to test the association of GRS_T2D_ with fetal weight and birthweight, adjusted for covariates, in each GD and GAP category.

**Results:**

Among women with the closest GD from African and Amerindigenous ancestries, the fourth and third GRS_*T2D*_ quartile was significantly associated with 5.18 to 7.48 g (weeks 17–20) and 6.83 to 25.44 g (weeks 19–27) larger fetal weight compared to the first quartile, respectively. Among women with middle GD from European ancestry, the fourth GRS_*T2D*_ quartile was significantly associated with 5.73 to 21.21 g (weeks 18–26) larger fetal weight. Furthermore, among women with middle GD from European and African ancestries, the fourth and second GRS_*T2D*_ quartiles were significantly associated with 117.04 g (95% CI = 23.88–210.20, *p* = 0.014) and 95.05 g (95% CI = 4.73–185.36, *p* = 0.039) larger birthweight compared to the first quartile, respectively. The absence of significant association among women with the closest GD from East Asian ancestry was complemented by a positive significant association among women with the highest East Asian GAP.

**Conclusions:**

The association between maternal GRS_*T2D*_ and fetal growth began in early-second trimester and was influenced by GD and GAP. The results suggest the use of genetic GD and GAP could improve the generalizability of GRS.

**Supplementary Information:**

The online version contains supplementary material available at 10.1186/s40246-024-00645-1.

## Background

Observational studies have shown an association between extremes of birthweight and an increased risk of perinatal morbidity and mortality, as well as long-term cardiovascular and metabolic complications such as type 2 diabetes (T2D) [1–4]. Genetic and non-genetic factors contribute to variations in fetal growth [5–9]. The maternal genome influences fetal growth through a direct impact on the intrauterine environment [8, 9], accounting for 7.6% of the genome-wide heritability of birthweight [9]. Previous studies, largely involving European ancestry individuals, showed that maternal genetic susceptibility to T2D contributes to increased fetal growth and birthweight [9–13]. It is known that the distribution of genetic variants, lifestyle factors, and their interactions can vary by ancestry [14, 15]. However, there is a knowledge gap in the link between maternal genetic susceptibility to T2D and fetal growth across different genetic ancestries.

The association between individual or aggregate effect (i.e., genetic risk score (GRS)) of genetic variants and health outcomes is poorly transferable across diverse ancestry groups [16]. GRS_T2D_ based on summary statistics from genome-wide association studies (GWAS) in African Americans was associated with increased birthweight in populations of African ancestry [13]. Likewise, GRS_*T2D*_ based on summary statistics from GWAS involving individuals of European ancestry showed a positive association with fetal weight beginning from 24 gestational week among European American women, but not among non-European American women [12]. The difference in GRS_T2D_ effects across the genetic ancestry continuum and the gestational weeks in which GRS_T2D_ begins to influence fetal growth are, therefore, unclear for most ancestral populations.

Addressing these research gaps through the use of self-reported race/ethnic groups or discrete genetic ancestry classifications has shortcomings [17]. For example, individuals categorized into the same ancestry group may exhibit variations along the genetic ancestry continuum. Consequently, it is challenging to establish clear boundaries between clusters, and some individuals may be excluded because of difficulty in assigning them to a specific ancestry group [17]. Moreover, the definitions of socially constructed race/ethnic groups and genetic ancestry are intricate and continuously evolving [18].

To address the aforementioned challenges, recent studies suggest using genetic distance (GD) [17, 19]. GD is a continuous metric that quantifies the similarity of an individual’s genome to the center of a standard reference population [17]. GD is considered a less biased estimator of genetic similarity as it takes into account inter-individual genetic variation within a given sample [17]. It has been shown that the accuracy and transferability of GRS derived from associations discovered in European GWAS decrease as the GD from a European ancestry reference population increases [17]. Additionally, the genetic ancestry proportion (GAP) of admixed populations affects the strength of the association between GRS and disease traits in Hispanic Americans [20]. Based on these recent developments, we posit that understanding the relationship between maternal genetic risk for T2D and fetal growth, accounting for GD and GAP, can provide insights about targeted intervention across different population groups. Therefore, in a cohort of multi-ancestral US pregnant women, we investigated the influence of maternal GD and GAP in the association between maternal GRS_T2D_ and fetal weight trajectories throughout gestational weeks 10–40.

## Methods

### Study population

Data from the *Eunice Kennedy Shriver* National Institute of Child Health and Human Development (NICHD) Fetal Growth Studies – Singletons cohort study, which recruited 2,802 women from four self-identified racial/ethnic groups (i.e., non-Hispanic white, non-Hispanic black, Hispanic white and Asian and Pacific Islanders), was used [21, 22]. The NICHD Fetal Growth Studies – Singletons cohort study was designed to develop a fetal growth standard for the US by race/ethnicity. Details on the inclusion criteria, data collection and quality assurance, and ethical approval were published elsewhere [21, 22]. Women completed sociodemographic, reproductive and pregnancy history questionnaires at enrollment in addition to providing blood specimen [21]. Clinical data was also extracted from medical records. The current analyses included 1,837 women with genotype data, at least two measures of fetal weight, and without gestational diabetes or hyperglycemia based on glucose challenge test.

### Fetal weight and birthweight measures

After ultrasonographic confirmation of gestational age at 8^+ 0^ and 13^+ 6^ weeks of gestation, participants were randomized to one of four groups and scheduled for five follow-up appointments to measure fetal growth biometrics using identical standardized obstetrical ultrasonography equipment and protocols [21, 22]. This randomization scheme was designed to capture all gestational week’s windows. Measurements between site sonographers and experts had a high correlation (> 0.99) and low coefficient of variation (< 3%) [23]. Fetal weight was estimated from head circumference, abdominal circumference, and femur length using Hadlock’s formula [24]. Fetal weight measures at gestational weeks 10–40 were estimated from the five measurements using a linear mixed model with a cubic spline mean structure and a cubic polynomial random effect [25]. Birthweight was measured in grams (g) using an electronic infant scale or beam balance scale [21].

### Genotyping and quality control

Genomic DNA was extracted from stored maternal blood specimens obtained from 2,215 (i.e., 641 self-identified non-Hispanic white, 652 non-Hispanic black, 582 Hispanic white and 340 Asian and Pacific Islander) women and genotyped using the Infinium Multiethnic Global BeadChip microarray (Illumina) with > 1.7 million single nucleotide polymorphisms (SNPs). A total of 2,056 women passed genotype quality control. SNP genotypes were imputed using the entire 1000 Genomes Phase 3 reference sequence data in the Michigan Imputation Server, implementing Eagle2 for haplotype phasing followed by Minimac3 for imputing non-typed SNPs. Details on genotyping, quality control and imputation procedures were described in our previous study [26].

### Genetic risk score for T2D (GRS_*T2D*_)

Publicly available summary statistics of 302 of the 338 genome-wide significant SNPs associated with T2D in the latest T2D multi-ancestry GWAS were used to calculate GRS_*T2D*_ (Supplementary Table 1) [27]. GRS aggregates the effects of genetic variants into a single score [28]. Weighted GRS_*T2D*_ was calculated by multiplying the dosage (i.e., values range 0 to 2) of the T2D-increasing allele for each SNP by logOR and the resulting values summed up [27]. We categorized the GRS_*T2D*_ into quartiles, and the lowest quartile (quartile 1) was used as a reference group.

### Genetic distance (GD) and ancestry proportion (GAP)

We used GD and ancestry proportion estimates to determine, for each woman’s genome, similarity with genotype of a reference population [29], and genome-wide average ancestry from each of four continental populations [30], respectively. First, genome-wide principal components analysis (PCA) of our cohort and the reference population (i.e., 1000 Genome and Human Genome Diversity Project population (HGDP)) genotype was performed using ‘*flashpcaR’* R package. Next, our cohort genotype was projected on the PC space of the reference population genotype. Unlike the previous studies that estimated GD using only European reference population [17, 19], we performed four types of GD estimations using three reference populations from the 1000 Genomes project [31]: European - Utah residents with Northern and Western European ancestry (CEU); African - Yoruba in Ibadan, Nigeria (YRI); and East Asian - Han Chinese in Beijing, China (CHB); and one reference population from the 1000 Genomes and Human Genome Diversity Project (HGDP) [32] – Amerindigenous – Native American (NAM). Then, GD of each woman’s genotype from the center of the reference population genotype projected on the PC space was calculated [17]. Finally, women were categorized into tertiles based on their GD and conventionally named as ‘closest GD’ (tertile 1), ‘middle GD’ (tertile 2) or ‘farthest GD’ (tertile 3) (Supplementary Table 2).

To supplement our findings based on GD, GAP was estimated for each sample by performing linkage disequilibrium pruning of the genotype data and running unsupervised clustering in ADMIXTURE version 1.3 software [30] with the assumption of four ancestral components (k = 4). These clusters represent European, African, Amerindigenous and East Asian genetic ancestry proportions. Reference samples for African, European and East Asian ancestries were obtained from genotype data of the 1000 Genomes project [31], whereas reference samples for Amerindigenous ancestry were obtained by combining samples from the 1000 Genomes and HGDP [31, 32]. The analyses were described in detail in our previous study [33]. Women were categorized into tertile based on their GAP and conventionally named as ‘lowest GAP’ (tertile 1), ‘medium GAP’ (tertile 2), and ‘highest GAP’ (tertile 3) (Supplementary Table 3).

### Data analyses

Maternal and newborn background characteristics were summarized using frequency (%) and mean (± SD). The correlation between GD and GAP was estimated using Spearman correlation method. Linear regression models were fitted to test the association of GRS_T2D_ with fetal weight at each gestation week (weeks 10–40) and birthweight, adjusted for years lived in the United States (continuous), marital status (categorical), maternal age (continuous), pre-pregnancy body mass index (continuous), parity (categorical), and fetal sex (categorical) in each GD and GAP category. The association between GRS_T2D_ and birthweight was further adjusted for gestational age at delivery (continuous). Additionally, interaction model was fitted by including interaction between GRS_T2D_ (continuous) and GD and GAP (continuous). Statistical significance was set to be p-value < 0.05. All analyses, unless specified otherwise, were implemented using the software package R version 4.2.2.

## Results

### Characteristics of study participants

The mean (± SD) of maternal age, number of years lived in the US, and birthweight were 28 (± 6) years, 23 (± 9) years, and 3,314 (± 529) grams, respectively. Approximately three-fourths of the women were married or living with a partner (73.54%). The mean (± SD) GRS_*T2D*_ was 16.19 (± 0.55). The mean (± SD) GD of the women from European, African, Amerindigenous, and East Asian reference panels was 0.03 (± 0.01), 0.03 (± 0.01), 0.13 (± 0.02), and 0.04 (± 0.01) unit, respectively. The mean (± SD) European, African, Amerindigenous, and East Asian GAPs in the maternal genome were 0.48 (± 0.37), 0.30 (± 0.36), 0.10 (± 0.20), and 0.12 (± 0.29), respectively (Table 1).

**Table 1:** Description of maternal and fetal background characteristics. (*N* = 1,837)

Characteristics	*n*(%) or Mean(± SD)
Maternal self-reported race/ethnicity	
Non-Hispanic white	562 (30.59)
Non-Hispanic black	573 (31.19)
Hispanic white	505 (27.49)
Asian/Pacific Islander	197 (10.72)
Maternal age (years)	27.88 (± 5.50)
Maternal birthplace, born in the United States	1,304 (70.99)
Years lived in the United States	23.03 (± 9.47)
Marital status, married or living with partner	1,351 (73.54)
Parity, ≥ one	980 (53.35)
Maternal pre-pregnancy BMI (kg/m^2^)	25.38 (± 5.13)
Gestational weight gain	12.41 (± 6.27)
Systolic blood pressure	109.94 (± 11.39)
Diastolic blood pressure	66.66 (± 8.95)
Gestational age at delivery (weeks)	39.16 (± 1.97)
Fetal weight (gram, g)	
Baseline	66.59 (± 14.71)
First visit	342.39 (± 172.85)
Second visit	1,075.02 (± 326.85)
Third visit	1,916.35 (± 407.51)
Fourth visit	2,749.93 (± 461.44)
Fifth visit	3,320.67 (± 469.94)
Birthweight (g)	3,313.92 (± 529.05)
Genetic risk score of type 2 diabetes (GRS_T2D_ )	16.19 (± 0.55)
Genetic distance from the reference panel	
From a European reference	0.03 (± 0.01)
From an African reference	0.03 (± 0.01)
From an Amerindigenous reference	0.13 (± 0.02)
From an East Asian reference	0.04 (± 0.01)
Genetic ancestry proportion	
European	0.48 (± 0.37)
African	0.30 (± 0.36)
Amerindigenous	0.10 (± 0.20)
East Asian	0.12 (± 0.29)
Fetal sex, male	857 (46.65)

**Fig. 1:**
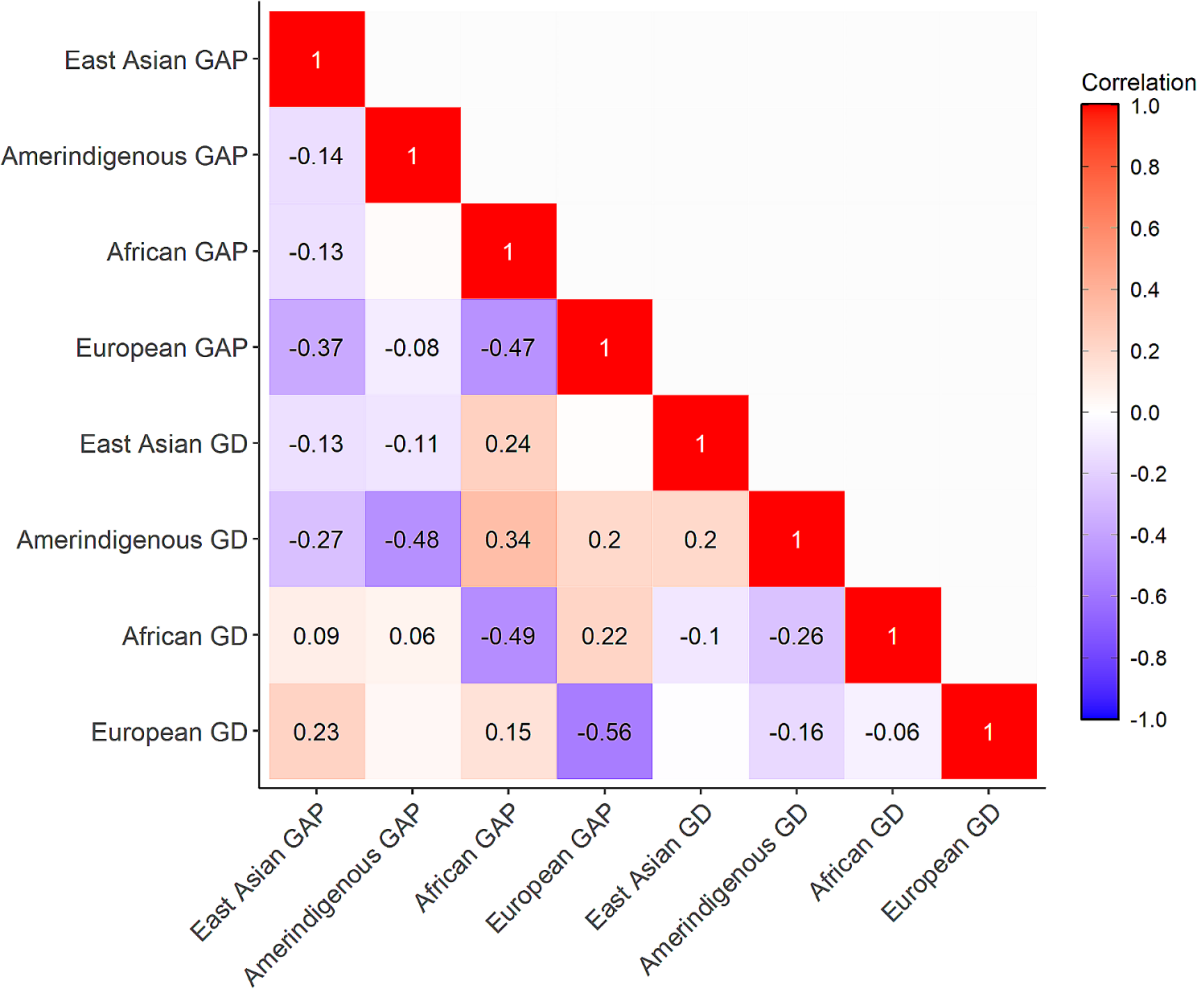
Correlation between genetic distance (GD) and genetic ancestry proportion (GAP). Numbers inside boxes denote correlation coefficients and are only shown for those with *p* < 0.05

There was weak to moderate negative correlation between GD from an ancestry reference and GAP of the same ancestry, for instance, with the lowest correlation between African GD and European GD (*r* = -0.0.06, *p* < 0.05) and the highest correlation between European GD and European GAP (*r* = -0.56, *p* < 0.05) (Fig. 1). Nearly all GD and GAP tertile groups included each of the four self-reported race/ ethnicity. No GD or GAP tertile represents less than three race/ethnicity groups, demonstrating the continuum nature of human population variation (Supplementary Figs. 1 and 2).

### GD and association of GRS_*T2D*_ with fetal weight

**Fig. 2:**
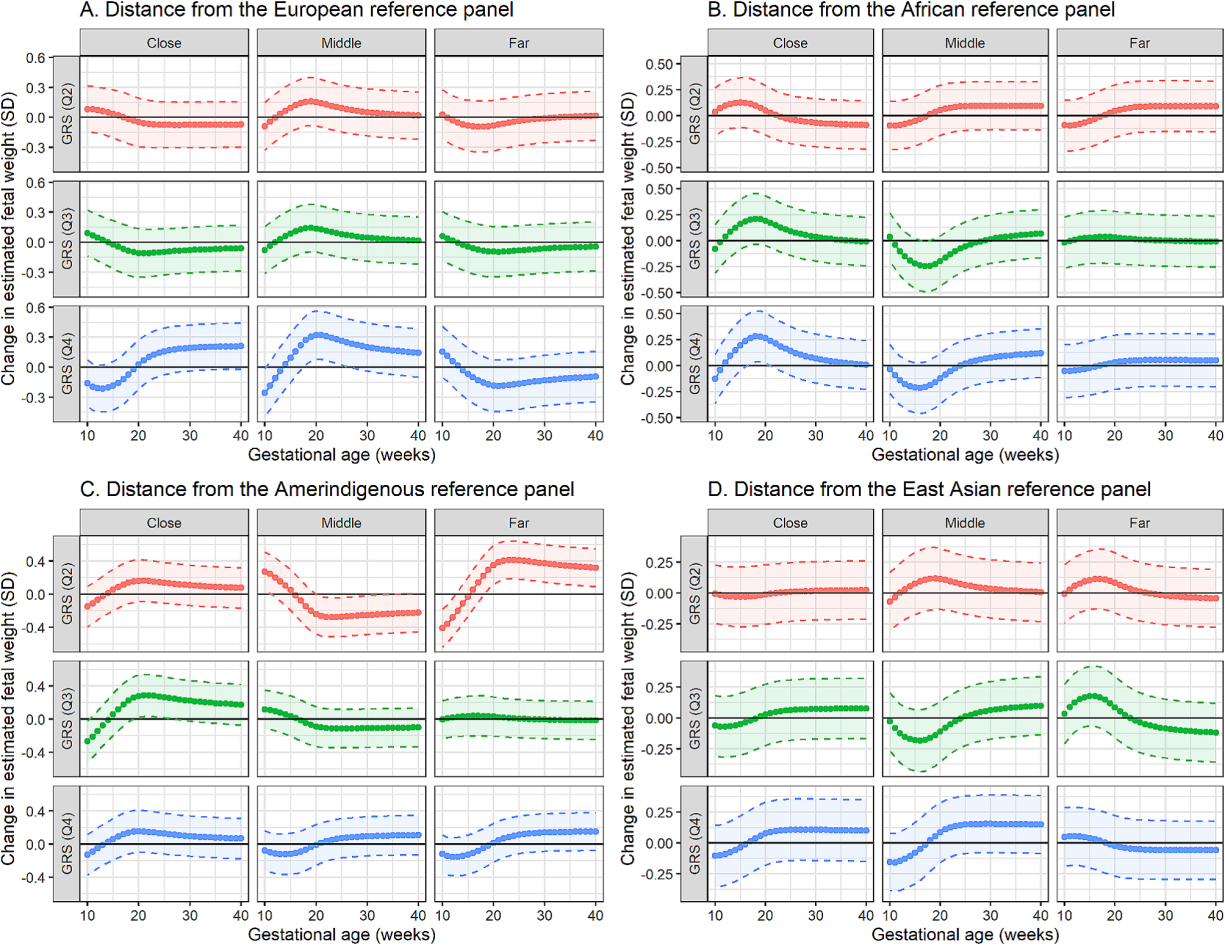
Weekly change in standardized fetal weight (in SD) associated with GRS_T2D_ based on GD. The dotted curved lines represent the average change in fetal weight, while the broken lines with colored zones indicate the 95% CI. The solid horizontal lines indicate the null hypothesis of no change in fetal weight. GRS (Q1) is the reference group

GRS_*T2D*_ showed significant positive association with fetal weight beginning from week 17 among women with the closest African GD, medium European GD, and irrespective of Amerindigenous GD. Specifically, the fourth GRS_*T2D*_ quartile was significantly associated with 5.73 to 21.21 g (weeks 18–26) and 5.18 to 7.48 g (weeks 17–20) larger fetal weight compared to the first quartile among women with the middle GD from European ancestry and with the closest GD from African ancestry, respectively. Among women with the closest and farthest GD from Amerindigenous ancestry, the third and second GRS_*T2D*_ quartiles were significantly associated with 6.83 to 25.44 g (weeks 19–27) and 7.65 to 149.71 g (weeks 19–40) larger fetal weight compared to the first quartile, respectively. Among women with middle GD from Amerindigenous ancestry, the second GRS_*T2D*_ quartile was significantly associated with 8.75 to 69.75 g (weeks 21–35) lower fetal weight. No significant associations were observed regardless of GD from East Asian ancestry (Fig. 2, Supplementary Fig. 3, Supplementary Table 4). The interaction model showed that the interaction between European GD and GRS_*T2D*_ significantly associated with fetal weight (weeks 21–40, *p* < 0.05).

### GD and association of GRS_*T2D*_ with birthweight

**Fig. 3:**
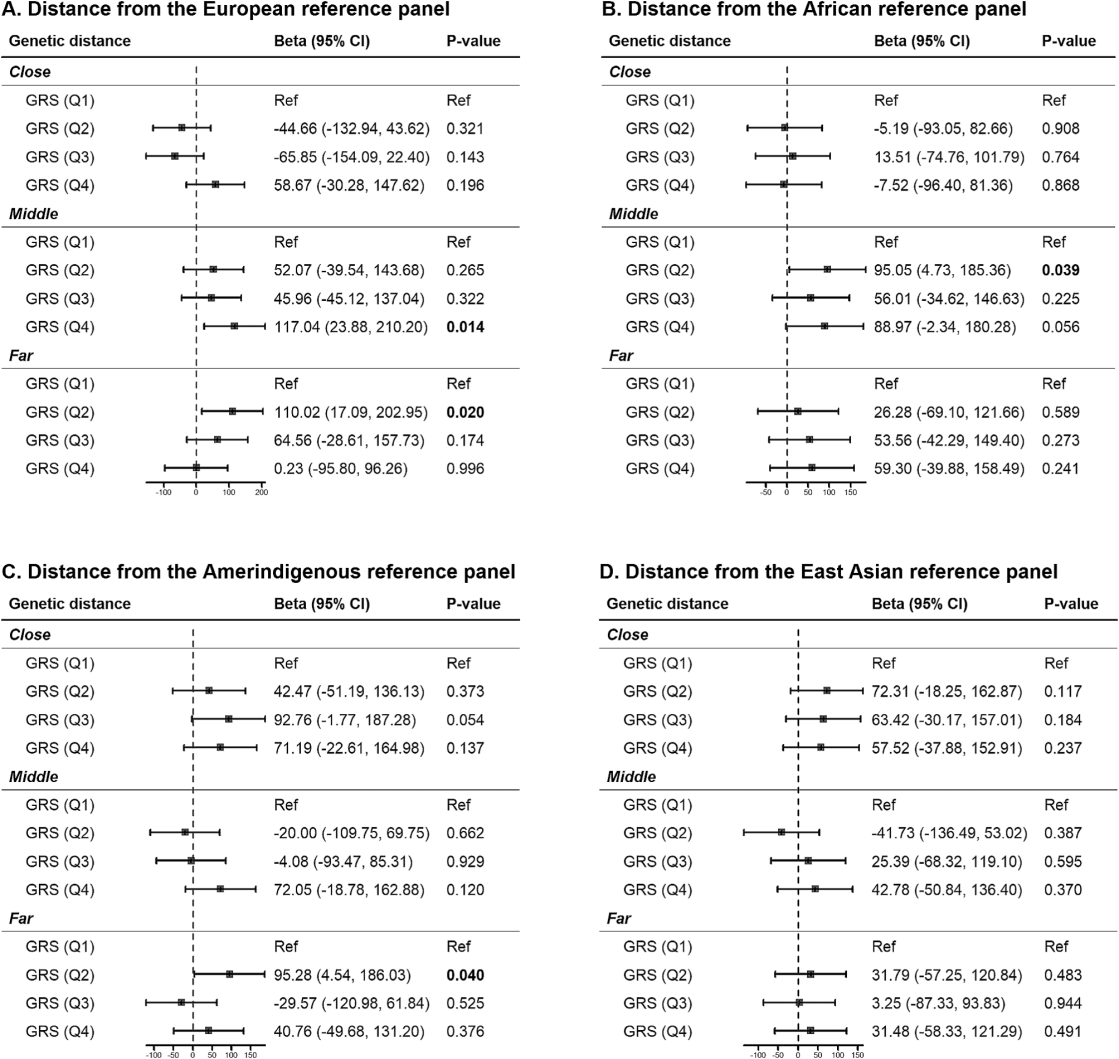
Change in birthweight (g) associated with GRS_T2D_ based on GD. The squares indicate the average change in birthweight, the horizontal lines represent the 95% CI, and the broken vertical lines indicate the null hypothesis of no change. GRS (Q1) is the reference group

GRS_*T2D*_ and birthweight showed significant positive association among women with medium and farthest European GD, medium African GD, and farthest Amerindigenous GD. Specifically, among women with middle and farthest GD from European ancestry, the fourth and second GRS_*T2D*_ quartile was significantly associated with a 117.04 g (95% CI = 23.88–210.20, *p* = 0.014) and 110.02 g (95% CI = 17.09–202.95, *p* = 0.02) larger birthweight compared to the first quartile, respectively. Compared to the first quartile, the second GRS_*T2D*_ quartile was significantly associated with a 95.05 g (95% CI = 4.73–185.36, *p* = 0.039) and 95.28 g (95% CI = 4.54–186.03, *p* = 0.04) larger birthweight among women with middle GD from African ancestry and with farthest GD from Amerindigenous ancestry, respectively. The interaction model showed that the interaction between European GD and GRS_*T2D*_ significantly associated with birthweight (*p* = 0.04). No significant association was observed regardless of GD from East Asian ancestry (Fig. 3).

### GAP and association of GRS_*T2D*_ with fetal weight

**Fig. 4:**
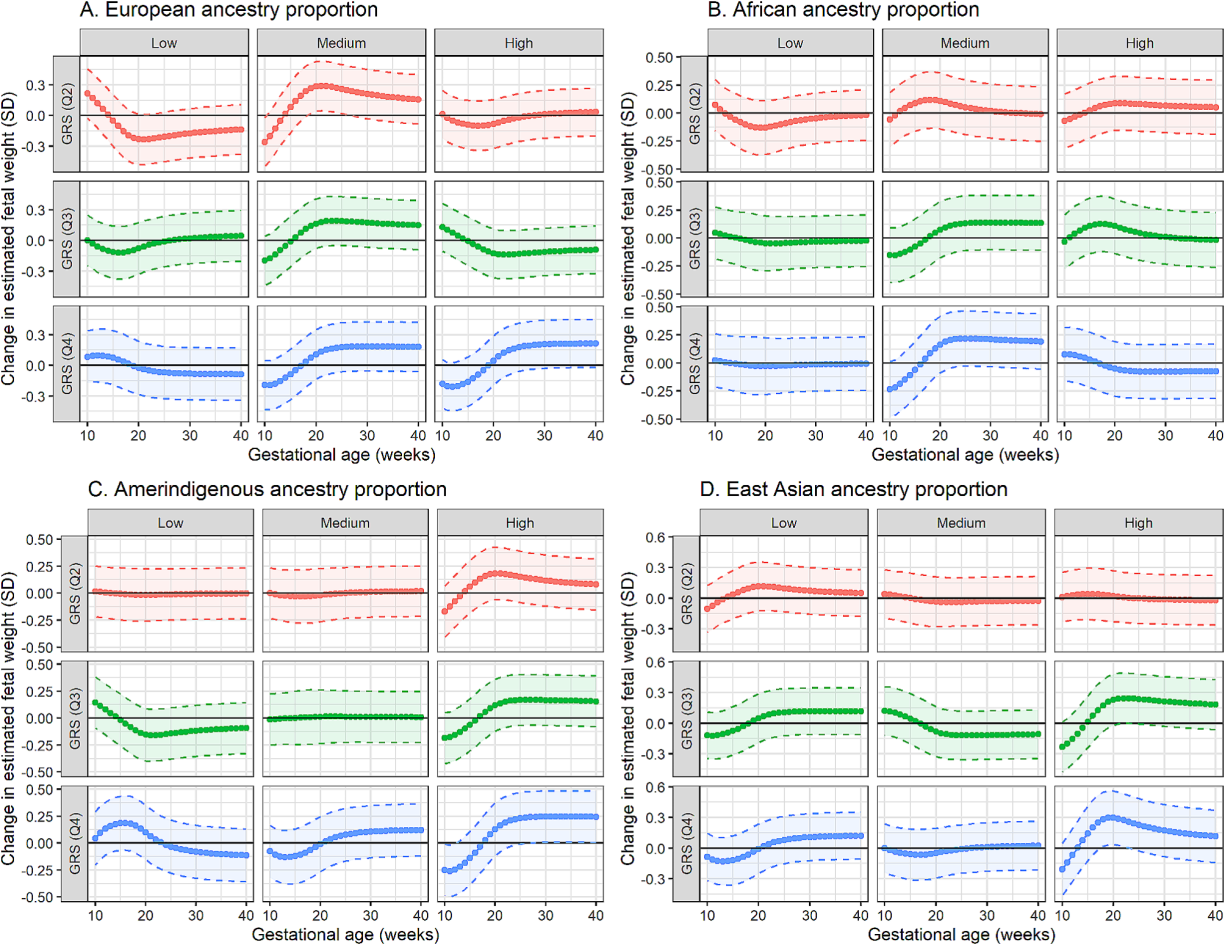
Weekly change in fetal weight (in SD) associated with GRS_T2D_ based on GAP. The dotted curved lines represent the average change in fetal weight, the broken lines with colored zones represent the 95% CI, and the solid horizontal lines represent the null hypothesis of no change in fetal weight. GRS (Q1) is the reference group

GRS_*T2D*_ showed significant positive associations with fetal weight among women with medium European GAP, highest Amerindigenous GAP, and highest East Asian GAP. Specifically, among women with medium European GAP, the second GRS_*T2D*_ quartile was significantly associated with 7.14 to 21.63 g (weeks 19–26) larger fetal weight compared to the lowest quartile. Among women with the highest Amerindigenous GAP, the fourth GRS_*T2D*_ quartile was significantly associated with 3.48 to 3.65 g (weeks 10–12) smaller fetal weight and 25.35 to 117.53 g (weeks 27–40) larger fetal weight. Among women with the highest East Asian GAP, the fourth GRS_*T2D*_ quartile was significantly associated with 5.93 to 13.26 g (weeks 18–23) larger fetal weight. No significant association was found regardless of African GAP (Fig. 4, Supplementary Fig. 4, Supplementary Table 5). The interaction model showed that the interaction between Amerindigenous GAP and GRS_*T2D*_ significantly associated with fetal weight (weeks 25–40, *p* < 0.05).

### GAP and association of GRS_*T2D*_ with birthweight

**Fig. 5:**
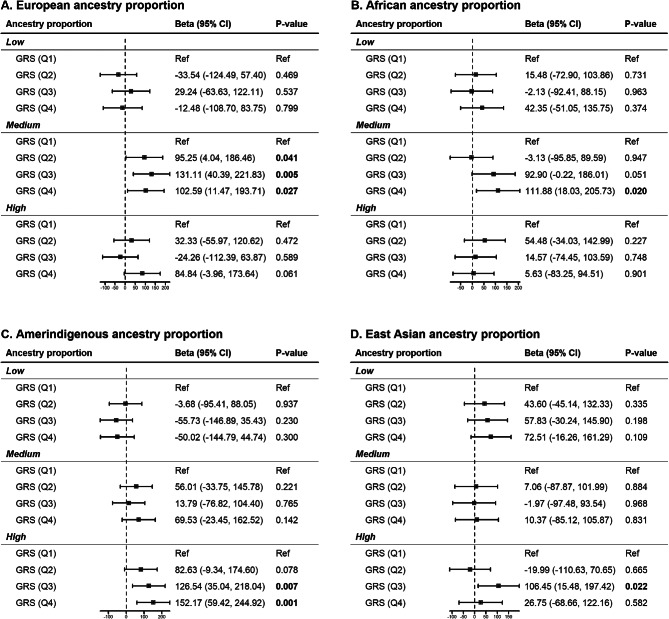
Change in birthweight (g) associated with GRS_T2D_ based on GAP. Squares represents the average change in birthweight, the horizontal lines represent the 95% CI, and the broken vertical lines represent the null hypothesis of no change. GRS (Q1) is the reference group

GRS_*T2D*_ and birthweight showed significant positive association among women with medium European GAP, medium African GAP, highest East Asian GAP, and highest Amerindigenous GAP. Specifically, among women with medium European GAP, the second, third, and fourth GRS_*T2D*_ quartiles were significantly associated with 95.25 g (95% CI = 4.04–186.46, *p* = 0.041), 131.11 g (95% CI = 40.39–221.83, *p* = 0.005), and 102.59 g (95% CI = 11.47–193.71, *p* = 0.027) larger birthweight compared to the lowest quartile, respectively. Compared to the lowest quartile, the fourth GRS_*T2D*_ quartile was significantly associated with 111.88 g (95% CI = 18.03–205.73, *p* = 0.02) among women with medium African GAP; and the third GRS_*T2D*_ quartile was significantly associated with 106.45 g (95% CI = 15.48–197.42, *p* = 0.022) larger birthweight among women with the highest East Asian GAP. Among women with the highest Amerindigenous GAP, the third and fourth GRS_*T2D*_ quartile was significantly associated with 126.54 g (95% CI = 35.04–218.04, *p* = 0.007) and 152.17 g (95% CI = 59.42–244.92, *p* = 0.001) larger birthweight, respectively (Fig. 5). The interaction model showed significant interaction between Amerindigenous GAP and GRS_*T2D*_ (*p* = 0.004).

## Discussion

By leveraging T2D-associated loci from a large multi-ancestral GWAS and incorporating genetic distance and ancestry proportion estimates in a multi-ancestral pregnancy cohort, we found that maternal GRS_*T2D*_ begins to influence fetal weight in early second trimester of pregnancy. Presence and gestational timing of associations between GRS_*T2D*_ and fetal weight varied by maternal GD and GAP. In many instances, the association between maternal GRS_*T2D*_ and fetal weight was influenced by both GD and GAP. For example, GRS_*T2D*_ was consistently associated with higher birthweight in women with middle or farthest GD from European, African, and Amerindigenous ancestries, as well as in those with the highest GAP. In other instances, the use of GAP yielded an association undetected when using GD. For example, while GRS_*T2D*_ was not associated with fetal weight or birthweight irrespective of GD from East Asian ancestry, significant association was detected with East Asian GAP. These findings and the weak to moderate correlation between GD and GA suggest the importance of considering both GD and GAP as alternative measures in identifying the timing and impact of GRS_*T2D*_ on fetal weight.

A key finding of our study is the detection of associations between maternal GRS_*T2D*_ and fetal weight in early second trimester (gestational weeks 17–19) based on GD from African, European, and Amerindigenous ancestry. In a previous study, maternal GRS_*T2D*_ was associated with increased fetal weight starting at week 24 among European American women, but not among non-European women [12]. In addition, maternal glucose levels measured at gestational weeks 10–14 have been significantly associated with fetal weight starting at week 27 [34]. The use of genetic distance estimates and implementation of GRS derived from a multi-ancestral GWAS may have facilitated our ability to identify a change in fetal weight at gestational weeks much earlier than previous studies.

There may be several factors that underlie the observed differences in the association of GRS_*T2D*_ with fetal growth by GD and GAP, especially after adjustment for maternal pre-pregnancy body mass index. Other cardiometabolic factors may have trajectories during pregnancy that differ across GD and GAP group, hence partly explaining these differences. For example, total gestational weight gain significantly differed across the continuum of Amerindigenous GD, systolic blood pressure differed across European and African GD, and diastolic blood pressure differed across European and Amerindigenous GD (Supplementary Table 2). Furthermore, out of the 302 SNPs included in the GRS calculation, 40% (121 out of 302) were associated with at least one cardiometabolic or anthropometric trait such as lipid profiles, blood pressure, adiposity and body composition, over-weight or obesity, and birth weight (Supplementary Table 1). Interactions among these factors and shared genetic effects could set the intrauterine metabolic, endocrine, and inflammation environment, potentially explaining differential effects of GRS_*T2D*_ on fetal growth by GD and GAP groups. Further investigation of the role of T2D-related SNPs in pregnancy-related cardiometabolic traits such as gestational weight gain, gestational diabetes, and gestational hypertension could provide additional insights into the association between GRS_T2D_ and fetal growth.

The mechanisms through which maternal T2D genetic risk induces fetal growth in early pregnancy are complex. A possible mechanism of the genetic effect is enhanced trans-placental glucose transfer which leads to increased secretion of fetal insulin, a growth hormone that promotes fetal growth [8, 9, 35]. Our finding is supported by this mechanism because fetal pancreatic release of insulin begins as early as 11 weeks of gestation [36]. Further studies should clarify the relationship between fetal insulin and glucose levels during early gestation. Moreover, non-glycemic pathways may underlie our observed associations because T2D shares genetic risk with metabolic and cardiovascular phenotypes, all of which are related to fetal growth [12]. Given the interdependent influence of the parental and fetal genomes on birthweight [8, 9], integrating fetal and parental genotype data will help elucidate the potential mechanisms underlying these associations [37–40].

Using multiple ancestry reference genotypes as anchors, we generated four GD estimates that allowed evaluation of GRS performance accounting for broad human population genetic variations. Previous studies tested the performance of GRS based on only GD estimates using a European ancestry reference genotype [17, 19], which could disregard the complex picture in human genetic variation and sociocultural differences. Using GD based on a single reference population could undermine predicting genetic influence in multi-ancestral populations that lead to increased heath disparity. For instance, although GRS_T2D_ was associated with fetal weight in the same gestational period for both the group with the closest GD from African and Amerindigenous references, the composition of those two groups by race/ethnicity is different. Even within a single ancestry group, the prediction accuracy of GRS may vary depending on factors such as socio-economic status, geographic distance, age, or sex of the individuals involved in the GWAS and prediction cohorts [15]. Moreover, race/ethnicity and genetic ancestry are complex constructs with evolving definitions due to the social nature of self-identified racial/ethnic constructs and phenotypic heterogeneity within broad racial/ethnic groups [12, 18]. Therefore, the use of multiple GD can partly overcome this limitation and lead to improved accuracy in prediction of genetic influence in non-European populations.

Although the GRS_T2D_ used in the present analysis was based on a T2D GWAS involving diverse populations, individuals of European ancestry form the vast majority of the study participants [27], which is a prevailing limitation of many genetic studies. Including ancestrally diverse individuals in genetic studies enhances insights into the genetic architecture of diseases [41]. Addressing the diversity gap in genomics is critical to ensure that all communities benefit from research innovations and precision medicine [42–44]. GRS derived from ancestry-matched and multi-ancestry studies will have better predictive accuracy, facilitating translation of genetic research into clinical care and public health policies that benefit a wide range of populations [45, 46].

Our study has several strengths. The GRS was derived from summary statistics of the largest T2D-GWAS in diverse ancestry groups [27]. The longitudinal ultrasound measures of fetal biometry in the NICHD Fetal Growth Studies allowed a comprehensive understanding of the influence of genetic variants on fetal growth at specific time points during pregnancy and at birth [21]. The study recruited low-risk pregnant women without previous history of major medical conditions and pregnancy complications, thereby minimizing potential confounding due to maternal morbidity [21].

We acknowledge certain limitations. The fetal effects of the SNPs used to calculate maternal GRS have not been accounted because fetal genotypes are unavailable in our dataset. Several studies consistently reported T2D-risk variants in the maternal and fetal genomes may have directionally opposite effects on insulin-mediated fetal growth [10, 47]. By design, women with type 2 diabetes were not enrolled in the study, limiting generalizability of the findings to women with glycemic dysregulations. Given the exploratory nature of our study, we did not apply multiple test correction. The Hadlock formula was used to calculate fetal weight, which may introduce inaccuracies particularly in the third trimester [24]. To address this issue, the average of three measurements was used, and the same sonographers were involved throughout the study period, minimizing intra-observer and inter-observer variability [23]. While GRS holds promise as a potential biomarker in medicine [48], it is crucial to validate its performance using studies with larger sample sizes and by integrating with non-genetic factors.

## Conclusions

Our study showed that the association between GRS_*T2D*_ and fetal growth begins in early second trimester of pregnancy and may vary depending on the reference population used to infer genetic similarity. Additionally, the use of both GD and GAP yielded valuable and alternative insights that may not be obtained when using either alone. The research question, study population, non-genetic factors, and their relationship with the chosen reference should be considered when selecting a more appropriate measure of genetic similarity. Further investigation is needed to evaluate the extent of maternal genetic susceptibility to T2D, integrated with other clinical and social determinants of health, can facilitate early detection of fetal growth changes, thereby mitigating adverse pregnancy outcomes and long-term diseases.

### Electronic supplementary material

Below is the link to the electronic supplementary material.

Supplementary Material 1

Supplementary Material 2

## Data Availability

The data sets generated and/or analyzed during the current study are available from the corresponding author upon reasonable request.

## Abbreviations

BMI: Body mass index
GAP: Genetic ancestry proportion
GD: Genetic distance
GRS: Genetic risk score
GRS_T2D_: Genetic risk score of type 2 diabetes
GWAS: Genome-wide association study
HGDP: Human genome diversity project
NICHD: National Institute of Child Health and Human Development
PCA: Principal component analysis
SD: Standard deviation
SNPs: Single nucleotide polymorphisms
T2D: Type 2 diabetes
