# Supplementary material for: Genetic distance and ancestry proportion modify the association between maternal genetic risk score of type 2 diabetes and fetal growth
Source: Hum Genomics. 2024 Jul 19;18:81. doi: 10.1186/s40246-024-00645-1 (PMC11264503; doi:10.1186/s40246-024-00645-1)

**Supplementary Figures**

**List of figures**

**Figure S1:** Distribution of self-reported race/ethnicity by genetic distance. This figure illustrates that even with just three genetic distance (GD) groups based on tertiles, there is no GD category that contains only women from a single self-reported race/ethnicity. Most GD categories include women from three or more self-reported race/ethnicities. The three GD tertile grouping is chosen to accommodate the limited sample size of the study for statistical power, yet it demonstrates continuity of human genetic variation that does not align with simple self-identified proxies. Categorization of GD into quintiles and deciles depicts this feature even more clearly.

**Figure S2:** Distribution of self-reported race/ethnicity by genetic ancestry proportion. This figure illustrates that even with just three genetic ancestry proportion (GAP) groups based on tertiles, there is a continuity of human genetic variation that does not align with simple self-identified proxies. GAP was determined in ADMIXTURE assuming four continental ancestry clusters. Hence further categorization of GAP into more categories would highlight this feature even more clearly.

**Figure S3**: Weekly change in fetal weight (in grams) associated with genetic risk score of type 2 diabetes based on genetic distance. The dotted curved lines represent the average change in fetal weight, while the broken lines with colored zones indicate the 95% CI. The solid horizontal lines indicate the null hypothesis of no change in fetal weight. GRS Q1 is the reference group.

**Figure S4**: Weekly change in fetal weight (in grams) associated with genetic risk score of type 2 diabetes based on genetic ancestry proportion. The dotted curved lines represent the average change in fetal weight, the broken lines with colored zones represent the 95% CI, and the solid horizontal lines represent the null hypothesis of no change in fetal weight. GRS Q1 is the reference group.

**Fig. S1:** Distribution of self-reported race/ethnicity by genetic distance. This figure illustrates that even with just three genetic distance (GD) groups based on tertiles, there is no GD category that contains only women from a single self-reported race/ethnicity. Most GD categories include women from three or more self-reported race/ethnicities. The three GD tertile grouping is chosen to accommodate the limited sample size of the study for statistical power, yet it demonstrates continuity of human genetic variation that does not align with simple self-identified proxies. Categorization of GD into quintiles and deciles depicts this feature even more clearly.


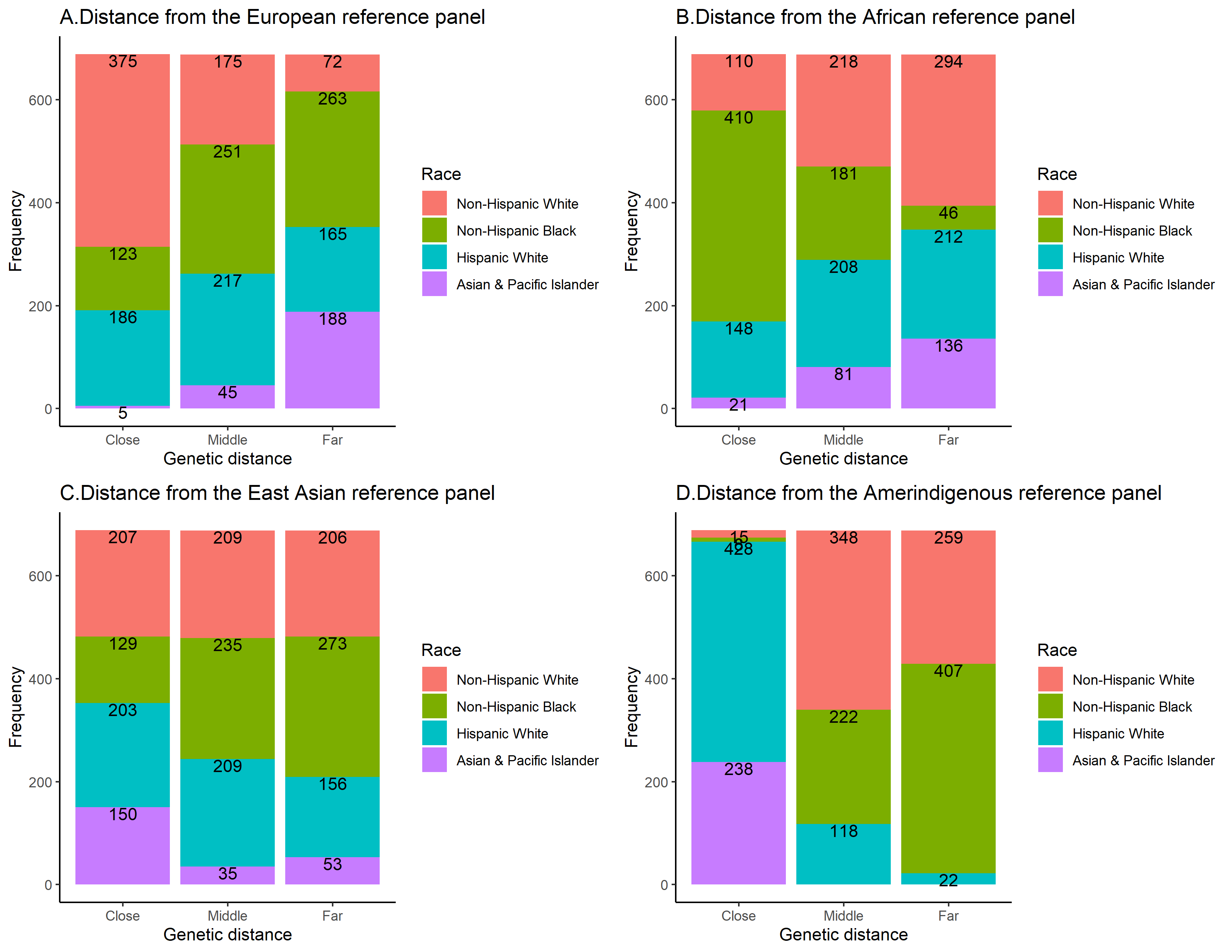


**Fig. S2:** Distribution of self-reported race/ethnicity by genetic ancestry proportion. This figure illustrates that even with just three genetic ancestry proportion (GAP) groups based on tertiles, there is a continuity of human genetic variation that does not align with simple self-identified proxies. GAP was determined in ADMIXTURE assuming four continental ancestry clusters. Hence further categorization of GAP into more categories would highlight this feature even more clearly.


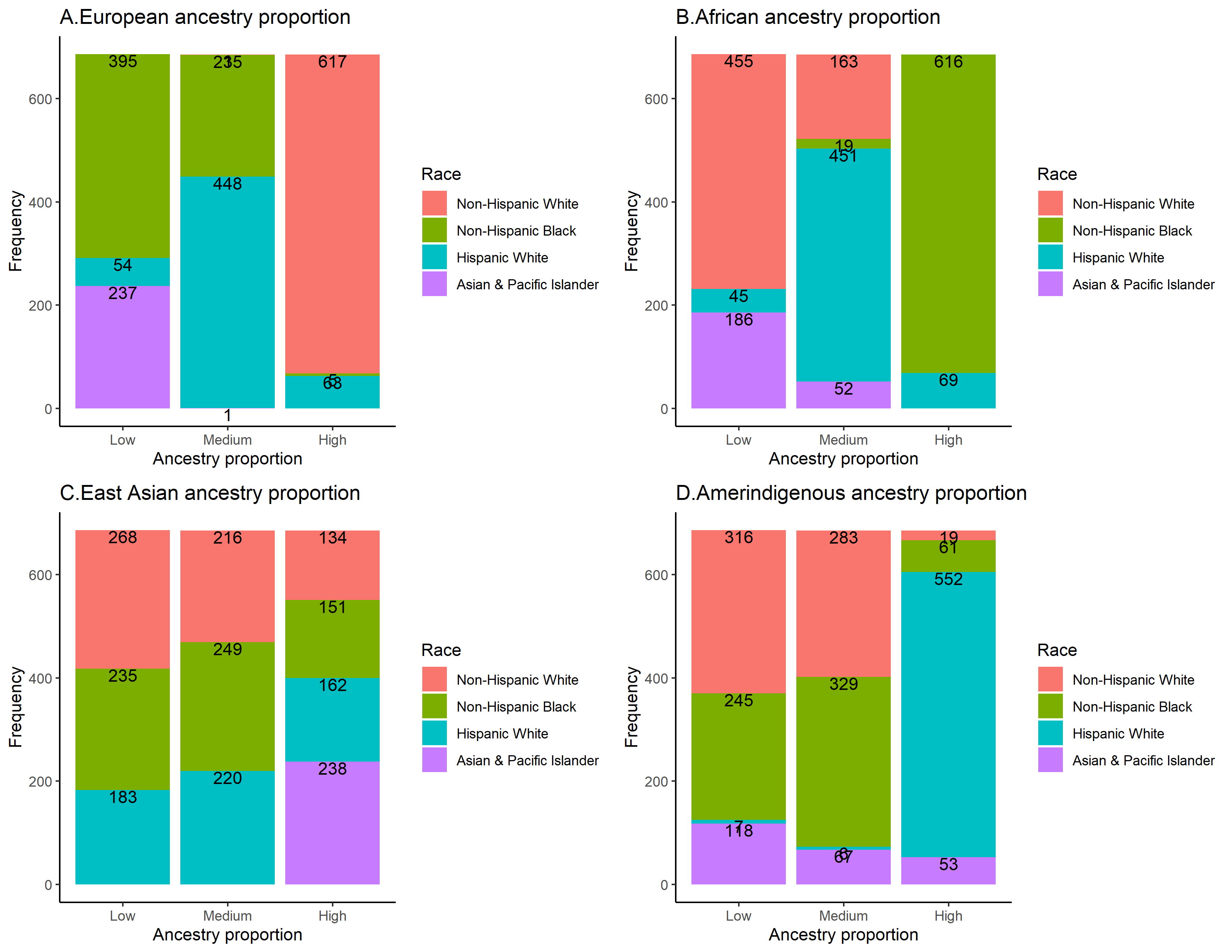


**Fig. S3**. Weekly change in fetal weight (in grams) associated with genetic risk score of type 2 diabetes based on genetic distance. The dotted curved lines represent the average change in fetal weight, while the broken lines with colored zones indicate the 95% CI. The solid horizontal lines indicate the null hypothesis of no change in fetal weight. GRS Q1 is the reference group.


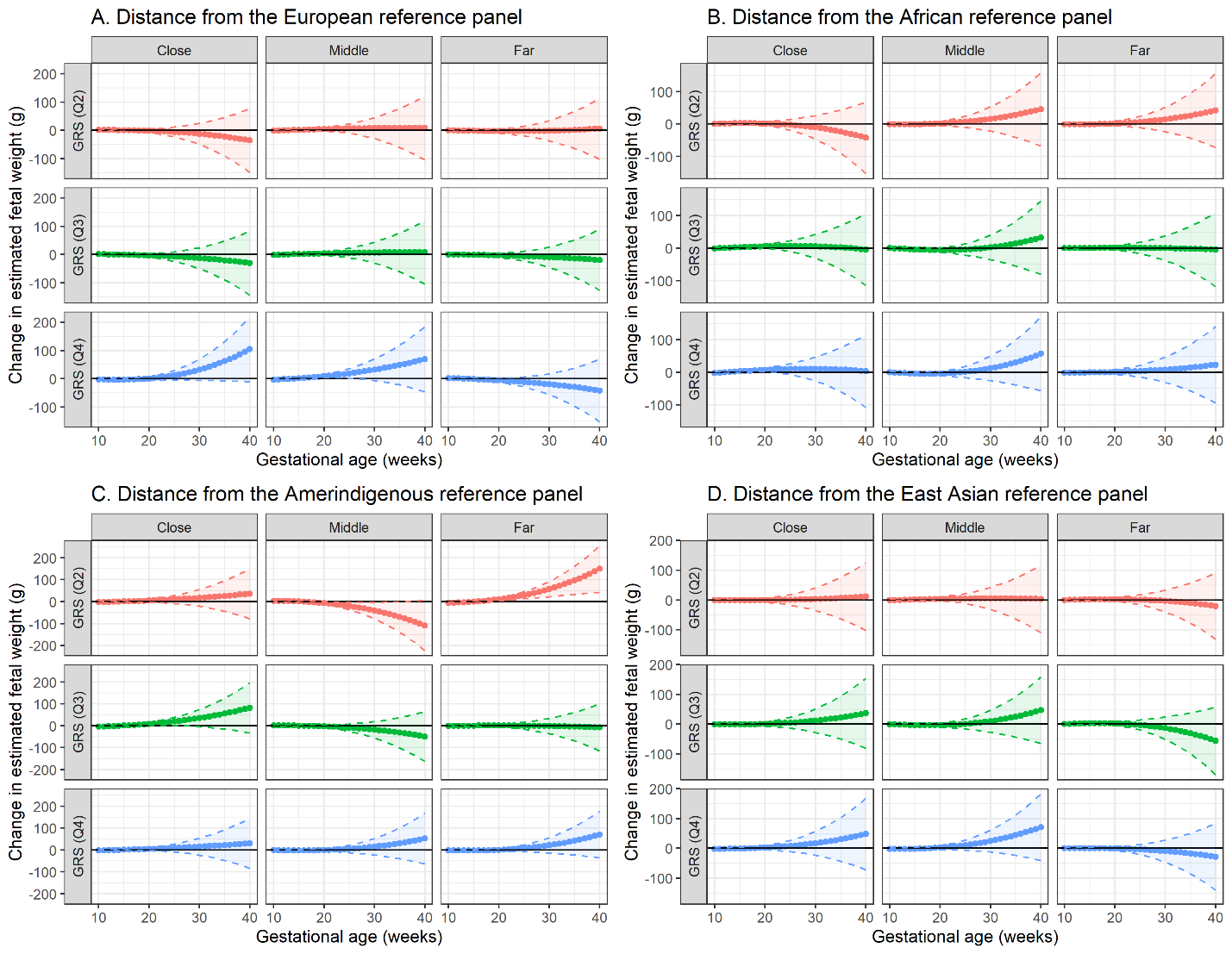


**Fig. S4**. Weekly change in fetal weight (in grams) associated with genetic risk score of type 2 diabetes based on genetic ancestry proportion. The dotted curved lines represent the average change in fetal weight, the broken lines with colored zones represent the 95% CI, and the solid horizontal lines represent the null hypothesis of no change in fetal weight. GRS Q1 is the reference group.


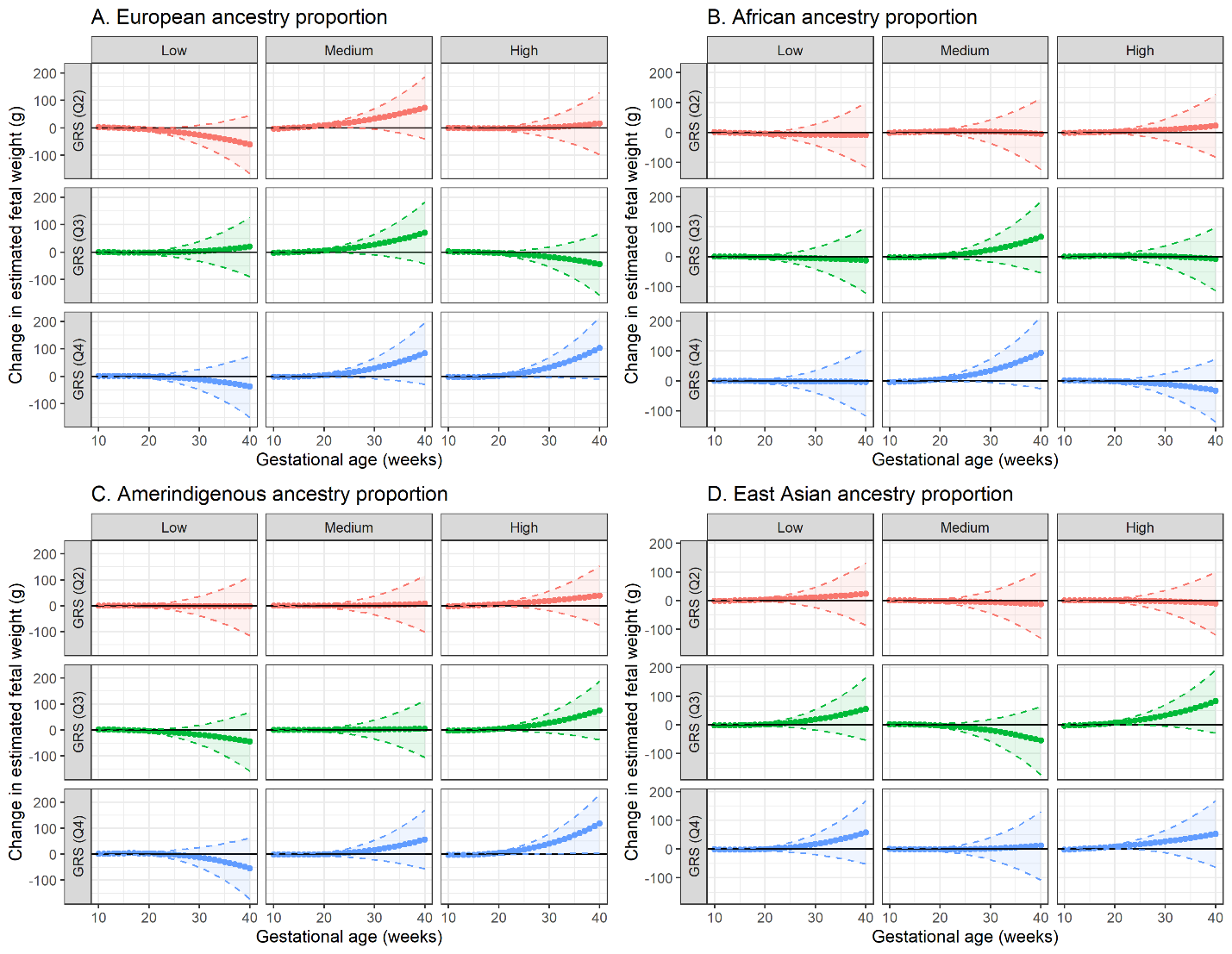

Supplement: Supplementary file 2 — Supplementary Material 2 [file 40246_2024_645_MOESM2_ESM.docx]
